# A Content Analysis of Nicotine Descriptors on the Front of Vape Packaging in the United Kingdom

**DOI:** 10.1093/ntr/ntae168

**Published:** 2024-07-25

**Authors:** Allison Ford, Anne Marie MacKintosh, Amber Morgan, Daniel Jones, Crawford Moodie, Kate Hunt, Kathryn Angus

**Affiliations:** Institute for Social Marketing and Health, University of Stirling, Stirling, UK; Institute for Social Marketing and Health, University of Stirling, Stirling, UK; Institute for Social Marketing and Health, University of Stirling, Stirling, UK; Institute for Social Marketing and Health, University of Stirling, Stirling, UK; Institute for Social Marketing and Health, University of Stirling, Stirling, UK; Institute for Social Marketing and Health, University of Stirling, Stirling, UK; Institute for Social Marketing and Health, University of Stirling, Stirling, UK

## Abstract

**Introduction:**

The Tobacco and Related Products Regulations (TRPR) 2016 require consumers in the United Kingdom to be informed about the presence of nicotine in vaping products. However, there is misunderstanding among some young people and adults around the strength of products. We examined how nicotine content is displayed on the front of vape packaging in the United Kingdom.

**Aims and Methods:**

Between August and December 2022, we systematically analyzed a representative, stratified selection of vapes and refill packs (*n* = 156) on the UK market to assess TRPR compliance. This paper presents an analysis of free-text responses collected to indicate the presence of nicotine information on the front-of-pack including metric, percentage, graphic, and text indicators. Data were analyzed using descriptive statistics produced for the sample as a whole and for five product categories.

**Results:**

Most packs (*n* = 126, 81%) displayed at least one front-of-pack nicotine descriptor, including the majority of disposables (*n* = 43, 90%), e-liquid (*n* = 42, 88%) and refill pods (*n* = 36, 100%). Many packs (*n* = 107, 69%) contained a nicotine-related metric (eg mg/ml), a quarter (*n* = 37, 24%) included a percentage indicator and most (*n* = 126, 81%) displayed at least one of these. Almost two-fifths (*n* = 57, 37%) mentioned nicotine beyond the warning. Less observed indicators included graphic and textual depictions of strength, dosage information, and an equivalent number of cigarettes.

**Conclusions:**

The front of vape packaging communicates important product information to consumers. There is inconsistency in how nicotine content is currently displayed. Future research should examine how best to display nicotine content to promote consumer understanding and informed decision making.

**Implications:**

This pack analysis of a representative sample of UK vape packaging highlights the varied ways in which nicotine content and strength are currently communicated to consumers on the front of vape packaging. The inconsistent presentation of nicotine content on the front of packs may contribute to misperceptions around product strength. A consistent and easily understood way of communicating nicotine content on the front of vape packaging may help consumers make more informed choices about vape products.

## Introduction

Many countries require vape (e-cigarette) companies to inform consumers about the presence of nicotine in nicotine-containing vaping products. In the United Kingdom, paragraph 37 of the Tobacco and Related Products Regulations (TRPR) 2016, concerning “Product information and labeling requirements,” stipulates that nicotine-containing vape packaging and refill containers must include (i) an indication of the nicotine content of the product, (ii) the delivery per dose, and (iii) a health warning covering 30% of the front and back of the pack with the text: “This product contains nicotine which is a highly addictive substance”.^1^ Our pack analysis of a representative sample of vape products sold in the United Kingdom in 2022 found good compliance across all-three items.^2^ We were lenient in judging indications of nicotine delivery dose being present, accepting this information being present anywhere on the pack. The lack of clarity in the Regulations for how a delivery dose should be indicated has been criticized.^3^

While it appears that companies are generally meeting these TRPR requirements, consumer perceptions around the nicotine content of vapes are problematic. A qualitative study with 16–70-year-olds with vaping experience in Great Britain found that many were confused about the nicotine strength of products.^4^ Misunderstanding was particularly evident in those using disposable vapes, who believed a nicotine descriptor of “2%” indicated low strength, despite this being the highest level of nicotine permitted in the United Kingdom (20 mg/ml).^4^ Similarly, adolescents and young adults in the United States rated concentrations presented as “mg/mL” as stronger, more addictive, and more harmful than equivalent concentrations presented in percentage form.^5^ These findings echo general public confusion, internationally, about nicotine.^6,7^

The front of consumer products’ packaging is a key vehicle for marketing and communications, particularly at the point-of-sale. While many studies have focused on how best to communicate nicotine addictiveness, eg to discourage vaping among adolescents while not discouraging adult smokers from switching to vaping,^8–11^ few studies have explored how nicotine content is displayed on vape packaging. Unlike the nicotine warning,^1^ there are no rules or guidance on this. We aimed to document the ways companies communicate nicotine content on the front of vape product packaging. We focus on the front-of-pack given its visibility and importance at the point-of-sale. Young people and adults highlight the lack of salience of messages placed elsewhere on the pack, particularly due to their smaller size.^2^ While others have examined the presence or absence of nicotine information,^12–14^ to our knowledge this is the first study to systematically examine the range of nicotine descriptors on the front of a representative sample of UK vape products’ packaging.

## Materials and Methods

### Data Sample and Coding

Data were collected between August and December 2022 as part of a larger study on the extent to which vapes and refills packaging complies with the TRPR, and how packaging is used as a promotional tool in the United Kingdom.^2^ Following a scoping of the UK vape market in land-based and online specialist stores and supermarkets, five product categories were sampled (e-liquids; disposable vapes; refill pods; tanks and cartomisers; and vape kits), and an online retailer with the most extensive coverage selected. Their stocklist was copied into Microsoft Excel, supplemented by other stores. For items available in different strengths (e-liquids, disposable vapes, and refill pods), products were subcategorized by nicotine strength to enable stratification. Nicotine-free versions, not covered in the TRPR, were excluded. From this spreadsheet (*n* = 3721), a minimum of 12 items in each product and strength category were randomly selected, and 156 unique products were purchased.

The codebook was informed by our previous study on e-cigarette advertising^15^; UK compliance guidance for e-cigarette manufacturers and retailers^3,12,16,17^; and research on packaging for e-liquids, tobacco, and food.^18,19^ The codebook collected 25 items covering whether the packaging requirements of the TRPR (mostly under Regulations 36–38) were met, and 29 items covering key packaging design and promotional features. Magnifying camera apps were used to view elements on packs. An SPSS database collected all codebook items using predefined and free-text responses. Ten percent of packs (*n* = 15) were double- or triple-coded as part of three successive pilots; the remainder of the sample (*n* = 141) was randomly allocated, stratified by product category, to KA and DJ for single-coding. Where the codebook had been amended after piloting, necessary updates were made to the previous coding. Half the single-coding was unblinded as one coder could view the first’s completed coding. Once finished, the database was reviewed together by KA and DJ, sorted by various items at a time (eg by product type or brand), to identify any divergences in coding that required rechecking for accuracy. AF checked (unblinded) a random 10% of each coder’s single-coded packs (covering all-five categories). Five authors reviewed the database to resolve coding discrepancies through discussion and agree on the dataset for analysis; see Moodie et al., 2023 (pp.78–83) for detailed sampling procedure, codebook design and data collection.^2^ This paper reports on the analysis of free-text data recorded as part of the original coding process (verbatim text and symbols displayed anywhere on the front-of-pack).

### Measures

Seven new measures recorded the presence (1) or absence (0) of information on the front of packs: (a) any mention of any metric (eg mg, mg/ml, mg/g); (b) any mention of “Nicotine” (beyond the warning) (eg “Nicotine” alongside a metric or percentage); (c) any inclusion of the term “percentage” or “percent” or “%”; (d) any graphic representation of strength (eg image of three rising bars with the first bar half-shaded); (e) any textual information relating to strength (eg High); (f) any information on dosage (eg Nicotine 506 µg per dose); and (g) any information equating the product to number of cigarettes (eg “equivalent to 20 cigarettes”). The number of nicotine descriptors on the front of pack was derived by summing the above measures with scores potentially ranging from 0 (no descriptors) to 7 (all-descriptor types present).

### Analysis

Data were analyzed using SPSSv29, with descriptive statistics produced for the sample as a whole and for each of the five product categories.

## Results

Table 1 shows the types of nicotine descriptors on the font of vape packaging. Most packs (*n* = 126, 81%) included at least one nicotine descriptor, including most disposables (*n* = 43, 90%), e-liquid (*n* = 42, 88%) and refill pods (*n* = 36, 100%). Three of the 12 tanks and cartomizers and two of the 12 vape kits contained any nicotine descriptors on the front of pack. Tanks and cartomizers and vape kits do not necessarily contain nicotine, but are capable of being used with nicotine.

**Table 1.** Nicotine Descriptors on the Front of Vape Packaging

Descriptors present on the front of pack												
	Total (*N* = 156)		Disposables (*N* = 48)		E-liquid (*N* = 48)		Refill pods (*N* = 36)		Tanks & Cartomizers (*N* = 12)		Vape kits (*N* = 12)	
	*n*	%	*n*	%	*n*	%	*n*	%	*n*	%	*n*	%
Any descriptors	**126**	**81**	43	90	42	88	36	100	3	[25]	2	[17]
Mention of “Nicotine” (beyond mention within the warning)	**56**	**36**	25	52	5	10	24	67	1	[8]	1	[8]
Metric and/or percentage (any mention)	**126**	**81**	43	90	42	88	36	100	3	[25]	2	[17]
With mention of nicotine	**56**	**36**	25	52	5	10	24	67	0	0	1	[8]
Without mention of nicotine	**70**	**45**	18	38	37	77	12	33	0	0	1	[8]
Metric (eg, mg, mg/ml, mg/g, etc) (any mention)	**107**	**69**	28	58	42	88	32	89	3	[25]	2	[17]
With mention of nicotine	**37**	**24**	10	21	5	10	20	56	1	[8]	1	[8]
Without mention of nicotine	**70**	**45**	18	38	37	77	12	33	2	[17]	1	[8]
Percentage (any mention)	**36**	**23**	28	58	3	6	4	11	0	0	1	[8]
With mention of nicotine	**28**	**18**	23	48	1	2	4	11	0	0	0	0
Without mention of nicotine	**8**	**5**	5	10	2	4	0	0	0	0	1	[8]
Metric & percentage both mentioned	**17**	**11**	13	27	3	6	0	0	0	0	1	[8]
Any other depiction of strength/dosage	**29**	**19**	1	2	10	21	17	47	0	0	1	[8]
Graphic depiction of strength	**23**	**15**	0	0	6	13	17	47	0	0	0	0
Text indicating strength	**5**	**3**	0	0	2	4	2	6	0	0	1	[8]
Dosage information	**2**	**1**	0	0	2	4	0	0	0	0	0	0
Information equating to the number of cigarettes	**1**	**1**	1	2	0	0	0	0	0	0	0	0

[] % is included for ease of comparison. Due to small base sizes, tanks & cartomizers and vape kits, %’s should not be quoted for these categories and actual numbers (*n*) should be used instead.

Overall, fewer than two-fifths (*n* = 56, 36%) of packs mentioned nicotine on the front-of-pack (beyond the warning, eg 20 mg salt nicotine, 1%/10 mg nicotine), although two-thirds (*n* = 24, 67%) of packs for refill pods and half (*n* = 25, 52%) of disposables did so.

Most packs (*n* = 107, 69%) contained some form of nicotine-related metric on the front-of-pack. In most cases (*n* = 88, 56%) this took the form of indicating “mg/ml”, while a few (*n* = 16, 10%) indicated “mg” and two cases indicated “mg/g”.

A quarter (*n* = 36, 23%) included a percentage indicator on the front-of-pack, including a majority of disposables (*n* = 28, 58%). A small number of e-liquids (*n* = 3, 6%), refill pods (*n* = 4, 11%) and one vapekit contained this. In most cases (*n* = 28), the percentage indicator was accompanied by the word “nicotine” (eg “2% nicotine”). On many packs (*n* = 17) it was accompanied by a metric (eg “2%/20 mg nicotine”, “2 ml 1.0%/10 mg/ml”). On a few packs (*n* = 3), the percentage indicator was accompanied by a text description of strength (eg “1.6% High Strength”).

Most packs (*n* = 126, 81%) contained either a nicotine-related metric or a percentage indicator. A small number (*n* = 17, 11%) contained both. Of these 126 packs, most (*n* = 70) did not mention nicotine alongside the metric or percentage indicator.

A minority (*n* = 23, 15%) provided a graphic depiction of strength, such as three rising bars or a range of solid dots with a maximum scale of five. This was only observed on e-liquids (*n* = 6, 13%) and refill pods (*n* = 17, 47%). There was no consistency in graphic depictions across brands. Few packs (*n* = 5, 3%) (two e-liquids, two refill pods, and one starter kit) indicated strength in text form (eg “Low”, and “High”).

Dosage information in the form of “12 mg/ml NICOTINE 506 µg PER DOSE” and “12 mg/ml NICOTINE 1988 µg PER DOSE” was indicated on two e-liquid packs from the same brand. One disposable vape pack indicated that the product was “Equivalent to 20 Cigarettes”.

Table 2 lists the number of nicotine descriptors present on the front of packs. On average, 1.47 (SD 0.99) nicotine descriptors were observed on the front of packs (Table 2), with refill pods and disposables averaging two, e-liquids averaging one, and the other products averaging fewer than one (Table 2).

**Table 2.** Number of Descriptors Present on the Front of Pack

Number of descriptors present on the front of pack												
	Total		Disposables		E-liquid		Refill pods		Tanks & cartomizers		Vape kits	
	*n*	%	*n*	%	*n*	%	*n*	%	*n*	%	*n*	%
None	**30**	**19**	5	10	6	13	0	0	9	[75]	10	[83]
One	**47**	**30**	13	27	29	60	3	8	2	[17]	0	0
Two	**56**	**36**	21	44	8	17	25	69	1	[8]	1	[8]
Three	**21**	**13**	9	19	5	10	6	17	0	0	1	[8]
Four	**2**	**1**	0	0	0	0	2	6	0	0	0	0
Mean	**1.47**		1.71		1.25		2.19		0.33		0.42	
SD	**0.99**		0.90		0.81		0.67		0.65		1.00	

[] % is included for ease of comparison. Due to small base sizes, tanks & cartomizers and vape kits, %’s should not be quoted for these categories and actual numbers (*n*) should be used instead.

## Discussion

This study documents the wide range of nicotine descriptors used on the front of UK vape packaging, highlighting the myriad ways (metric, eg mg, mg/ml, mg/g; percentage; graphic and text indication of strength; dosage information; equivalent number of cigarettes) companies communicate nicotine content and strength. Around one-third of packs used the word “nicotine” alongside a descriptor. The number of descriptors displayed ranged from none to four.

While companies are technically fulfilling current TRPR requirements to provide nicotine information on vape products’ packaging,^2^ the lack of consistency in *how* companies communicate nicotine content on the front of vape packaging may be contributing to consumers’ confusion around the strength of nicotine-containing vapes.^4,5^ Our study found many packs displayed a metric and/or percentage, without mentioning nicotine, potentially making consumer interpretation difficult. Additional information such as “number of puffs” (we noted such text on around two-thirds of disposables) may also add to misunderstanding. More specific rules on the display of nicotine content may benefit consumers, alongside clear guidance for companies. Detailed consumer research, for example, with young people and adults, nicotine and non-nicotine users, could provide valuable evidence to regulators on how best to communicate product strength. A clear understanding of nicotine descriptors and strength, including the maximum legally allowed, enables consumers to make more informed choices. Research must explore consumer understanding of nicotine and which descriptors and/or symbols are most easily understood. To date, research has focused on the communication of nicotine as an addictive substance on vape packaging,^8–11^ yet evidence is mixed on how nicotine addiction warnings affect harm and addictiveness perceptions, and intentions to vape. It is important to assess the impact of nicotine descriptors on these perceptions and intentions.

Within our pack sample, nicotine content displayed in percentage form was mostly found on disposable vapes’ packaging. Given the misperception among some vapers that a “2%” descriptor indicates low strength,^4,5^ it may be that in the absence of an understanding of nicotine descriptors, consumers use other cues on the pack to inform their perceptions. Within vape packaging, the presence of bright colours, cartoonish scripts and crayoned fonts,^2^ may contribute further to misperceptions around nicotine strength. In March 2024, a Tobacco and Vapes Bill was introduced in the UK Parliament to address the recent rise in youth vaping in the United Kingdom and reduce the appeal of vapes to young people, giving the government power to regulate vape packaging.^20^ Further consultation will explore specific measures; however, this presents an opportunity to standardize product information in the most helpful way to consumers.

Our study benefits from a representative sample of vape products’ packaging legally sold in the UK, extending the literature beyond the presence or absence of nicotine information,^12–14^ to document how nicotine content is displayed across vape products. We note some limitations. Most packs were single-coded, although checks were made throughout the coding process and consensus on discrepancies was reached through discussion. We focused on the front-of-pack given its importance for marketing and communication. Further descriptors may be printed elsewhere or within leaflets. The packs may not represent vape products sold in other jurisdictions, although many brands sampled were international and may use the same descriptors and shorthand “symbols.”

## Conclusion

Vape packaging communicates product information to consumers and potential consumers. Our findings highlight the lack of consistency in how nicotine content is displayed on the front of UK vape packaging. This may have implications for consumer understanding of product strength. Future research should explore how best to display nicotine content, so the strength of products is easily understood by consumers.

## Data Availability

The data that supports the findings of this study are available from the corresponding author, upon reasonable request.
